# Prediction of 1-year post-operative mortality in elderly patients with fragility hip fractures in China: evaluation of risk prediction models

**DOI:** 10.3389/fsurg.2025.1415680

**Published:** 2025-06-23

**Authors:** Qiyuan Lu, Mengmeng Chen, Houfu Ling

**Affiliations:** ^1^ Department of Orthopaedics, Yuyao Hospital of Traditional Chinese Medicine, Ningbo, Zhengjiang, China; ^2^ Department of Orthopaedics, Cixi Third People's Hospital, Ningbo, Zhengjiang, China; ^3^ Department of Orthopaedics, The First Affiliated Hospital of Zhejiang Chinese Medical University (Zhejiang Provincial Hospital of Chinese Medicine), Hangzhou, Zhejiang, China

**Keywords:** fragility hip fracture, mortality, risk prediction, risk stratification, NHFS

## Abstract

This study systematically evaluates the predictive capacity of seven risk stratification models for 12-month postoperative mortality in geriatric patients with fragility hip fractures, while concurrently assessing their risk classification accuracy to inform perioperative protocol formulation, rehabilitation strategies, and prognostic management.

**Introduction:**

Current clinical practice lacks standardized criteria for mortality risk prediction in elderly fragility hip fracture patients. This investigation conducts a comparative evaluation of seven prognostic models—the Sernbo Score, Jiang et al. model, Nottingham Hip Fracture Score (*N*HFS), Holt et al. algorithm, HEMA, ASAgeCoGeCC Score, and SHiPS—HiPSe, and SHire, and SHim, HEMA, ham Hip Fracture Score (mortality risk prediction in elderly fragility hip fracture patients

**Methods:**

In this retrospective cohort analysis, all consecutive patients aged isk prediction in elderly fragility hip fracture between January 2018 and October 2022 were enrolled. Model-derived mortality predictions and risk categorizations were computed. Predictive performance was quantified through the predictive validity, the area under the receiver operating characteristic (ROC) curve (AUC) analysis, DeLong test, Hosmer-Lemeshow goodness-of-fit testing and calibration slope (95% CI), followed by precision assessment of risk stratification tiers.

**Results:**

The cohort demonstrated a 12-month mortality rate of 29.0%. Kaplan–Meier survival curves identified the first postoperative year as the highest mortality risk period. The ASAgeCoGeCC Score was the only model in this study that simultaneously demonstrated balanced sensitivity (0.73)/specificity (0.82), excellent discrimination (AUC = 0.84), and good calibration (H-L *p* = 0.36, calibration slope = 0.75). The DeLong test indicated its significantly superior performance compared to the other models (*p* < 0.01). The NHFS and Holt et al. performed next best. All models except the Sernbo Score achieved AUC values exceeding 0.70. Significant calibration deficiencies were observed in NHFS, HEMA, and SHiPS (Hosmer-Lemeshow *p* < 0.05). Risk stratification analysis revealed SHiPS as the most precise classification system.

**Conclusion:**

ASAgeCoGeCC score, NHFS and Holt et al.showed acceptable predictive performance, where the first two are applicable to clinical rapid decision-making, while NHFS has been extensively external validated. Holt et al.is more suitable for a well-resourced medical system. SHiPS displayed optimal risk categorization accuracy, suggesting potential for broader clinical implementation. These findings necessitate verification through prospective multi-center studies.

## Introduction

Osteoporotic fractures, clinically designated as fragility fractures, constitute skeletal injuries resulting from low-energy trauma (equivalent to a fall from standing height or less) and represent a critical manifestation of advanced osteoporosis (1). With the progressive aging of the global population, these fractures now account for 34.8% of the global disease burden attributed to non-communicable pathologies (2). Epidemiological data from China demonstrate a marked escalation in osteoporotic fracture prevalence, rising from 13.2% during 2000–2010 to 22.7% in the 2012–2022 period (3). National projections indicate a rise from 2.33 million documented cases in 2010 to an anticipated 5.99 million by 2050 (1, 4).

As the most prevalent subtype of fragility fractures, hip fractures significantly impair functional independence while imposing substantial socioeconomic burdens through extended care requirements (3, 4). The Chinese population exhibits particularly concerning outcomes, with fragility hip fractures demonstrating elevated disability rates and mortality indices (3). Comparative analyses reveal a ninefold increase in mortality risk relative to the general population, with first-year mortality rates among elderly patients ranging from 16.5% to 33.0% (3, 5). Concurrently, projected healthcare expenditures for osteoporotic fracture management are anticipated to escalate from ¥69 billion (2010) to ¥163 billion by 2050.

Current clinical guidelines advocate surgical intervention for geriatric hip fracture patients without severe comorbidities. However, this population presents unique challenges including diminished bone mineral density, elevated perioperative risk profiles, and suboptimal postoperative outcomes. Risk stratification models demonstrating robust predictive validity could enhance clinical decision-making through mortality risk quantification and prognostic forecasting. Notwithstanding the development of multiple predictive instruments, including the validated Nottingham Hip Fracture Score (NHFS) for short-term mortality prediction, significant limitations persist. Most novel models remain in external validation phases, with indeterminate predictive capacity for long-term mortality in elderly fragility hip fracture patients. Notably, no dedicated predictive tool currently exists for this high-risk demographic.

This study aims to conduct a comprehensive evaluation of existing mortality prediction models regarding their: (1) prognostic accuracy for 12-month mortality in elderly fragility hip fracture patients, (2) risk stratification reliability, and (3) clinical applicability. The findings are anticipated to inform evidence-based clinical practice across healthcare institutions managing geriatric fragility hip fractures, ultimately contributing to reduced one-year mortality rates.

## Patients and methods

### Data sources

This retrospective cohort study included elderly patients with fragility hip fractures who were hospitalized in Zhejiang Provincial Hospital of Traditional Chinese Medicine from January 2018 to October 2022, and allowed at least one year of follow-up.

The following keywords were searched in the hospitalization system: “femoral neck fracture”, “intertrochanteric fracture”, “subtrochanteric fracture”. Dual energy x-ray absorptiometry (DXA) was performed in all hip fracture patients over 55 years of age. Patients with a T-value from DXA > −2.5 are excluded from the study.

Patients with conservative treatment, periprosthetic femoral fracture, pathological fracture or no osteoporosis were excluded. Surgical treatment is in line with China's guidelines. A total of 7 risk prediction models for mortality were evaluated.

### Risk prediction model

This study selected commonly used hip fracture prediction models through studies and conducted external validation in elderly patients with fragility hip fractures who underwent surgical treatment at our institution, aiming to evaluate the applicability and accuracy of these models for this specific population.

A total of seven mortality risk prediction models were included, provided that complete data for all required variables were available. Models such as NHFS and HEMA (Hip fracture estimator of mortality Amsterdam) were specifically designed for hip fracture patients, with variable selection tailored to elderly population characteristics. The Sernbo Score, developed using simple indicators like walking ability and living status to predict survival, is suitable for rapid bedside assessment. SHiPS (Shizuoka Hip Fracture Prognostic Score) and ASAgeCoGeCC are integer-based scoring systems with clearly defined variable weights, enabling straightforward risk assessment without complex calculations. Studies of Jiang et al. and Holt et al., often based on retrospective cohorts, prioritize easily accessible variables for direct clinical implementation. Detailed scoring criteria for each model are summarized in Table 1.

**Table 1:** Seven death risk prediction models name, variables, value and score.

Risk model/ Variables	Value	Score
Sernbo score		
Age	<80 years	5
	≥80 years	2
Habitat	Own home	5
	Sheltered home or frequent home assistance	2
Walking aids	None, or one stick	5
	Two sticks or walking frame	2
Mental status	Alert	5
	Slight confusion	2
Jiang et al.		
Age	60–69	0
	70–79	6
	80–89	7
	≥90	13
Male sex		6
Admitted from longterm care		4
COPD		4
Pneumonia		14
Ischemic heart disease		5
Previous myocardial infarction		13
Any cardiac arrhythmia		5
Congestive heart failure		7
Malignancy		13
Malnutrition		20
Any electrolyte disorder		5
Renal failure		19
NHFS		
Age in years	<66	0
	66–85	3
	≥86	4
Sex	Male	1
Admission Hb	≤10 g dl^−1^	1
Cognitive impairment	Yes	1
Living in an institution	Yes	1
Number of co-morbidities	≥2	1
Malignancy	Yes	1
Holt et al.		
Age in years	<60	0
	60–69	0.58
	70–79	1.24
	80–89	1.74
	≥90	1.96
ASA score	1 or 2	0
	3	0.80
	4 or 5	1.62
Gender	Male	0
	Female	−0.65
Pre-fracture residence	Own home	0
	Long-term care	0.53
	Rehabilitation	0.53
	Acute hospital ward	0.59
Pre-fracture mobility	No aids	0
	One aid	−0.02
	Two aids/frame	0.07
	Requires accompaniment	0.24
	Unable to walk	0.45
Fracture type	Intracapsular	0
	Extracapsular	0.12
	Subtrochanteric	0.28
	Pathological	1.32
HEMA		
Age in years	≥85	1
In-hospital fracture	Yes	2
Signs of malnutrition	Yes	2
Myocardial infarction	Yes	1
Congestive heart failure	Yes	1
Current pneumonia	Yes	2
Renal disease	Yes	1
Malignancy	Yes	1.5
Serum urea	>9 mmol/L	0.5
ASAgeCoGeCC Score		
Age	65–74	0
	75–85	1
	>85	2
CCI	3–4	3–37
	>4	
Cognitive impairment	No	0
	Yes	1
ASA	<3	0
	3	1
	>3	2
Gender	Female	0
	Male	1
SHiPS		
Sex	Male	8
	Female	0
Age	65–74	0
	75–84	5
	85–94	10
	≥95	16
Fracture site	Fracture of femoral neck	1
	Pertrochanteric fracture	1
	Subtrochanteric fracture	0
Nursing care certification	Yes	8
Comorbidity	Any malignancy	
	Metastatic solid tumor	9
	Other malignancy except neoplasm of skin	3
	Malignant neoplasm of skin	0
	Moderate or severe liver disease	8
	Renal disease	4
	Congestive heart failure	3
	Deficiency anemia	2
	Chronic pulmonary disease	1

NHFS, Nottingham Hip FractureScore; HEMA, Hip fracture estimator of mortality Amsterdam; SHiPS, Shizuoka Hip Fracture Prognostic Score.

### Sernbo score

The Sernbo Score was originally developed as a tool for decision-making in the treatment of femoral neck fractures. It consists of four variables: Age, Habitat, Walking aids and Mental status. Each variable scored 2 or 5 points, and the total score was 8, 11, 13, 17 or 20 points. For displaced fractures of the neck of the femur, total hip arthroplasty should be performed if the total score is more than 15 points, and hemiarthroplasty should be selected if the total score is less than 15 points (6). Three empirical subgroups were formed: low risk (17 or 20 points), moderate risk (14 points), and high risk (8 or 11 points) (7).

### Jiang et al

Jiang et al. is a multivariate risk-adjusted model based on comorbidity in patients with hip fractures to predict 30-day and 1-year mortality in such patients. It consists of four parts: age, gender, long-term care residence and comorbidity, among which comorbidity includes 10 different diseases (8). The score of each variable was between 0 and 20, and the predicted probability of in-hospital death was from <1% to >15%.

#### NHFS

NHFS was validated as a predictor of 30-day and 1-year mortality following surgically managed neck of femur fractures. Subsequently, the model showed good predictive performance for hip fractures, periprosthetic fractures, etc. in external validation. NHFS was developed by Maxwell et al. in 2008 and recalibrated after longitudinal evaluation in 2012 to correct the overestimation of mortality in the high-risk group (9). NHFS was composed of seven variables: age, gender, serum haemoglobin, Abbreviated Mental Test Score (AMTS), whether the patient is living in an institution, the number of comorbidities and a history of malignancy. The predicted 30-day mortality was calculated using the formula 100/1 + *e*^(5.0122−(NHFS*0.481))^. Due to the retrospective characteristics of this study, patients did not perform AMTS score at admission, so we used a history of cognitive impairment to replace AMTS.

### Holt et al

Holt et al. was developed to predict 30-day and 120-day mortality after hip fracture surgery. The predictive model proposed by Holt et al. included six variables: age, American Society of Anesthesiologists (ASA) score, gender, pre-fracture residence, pre-fracture mobility and fracture type to predict 30-day and 120-day mortality after hip fracture surgery (10). The predicted 30-day and 120-day mortality rates were calculated using the formula mortality = 1/1 + *e*^−(constant^ ^+^ ^B (ASA)^ ^+^ ^B (pre-fracture residence)+B (age)^ ^+^ ^B (sex)^ ^+^ ^B (type of fracture)^ ^+^ ^B (pre−fracture mobility)^.

#### HEMA

HEMA was originally designed to predict 30-day mortality after hip fracture surgery and to identify patients requiring more intensive perioperative care. In 2018, Karres et al. developed a HEMA score based on nine variables: age, in-hospital fracture, signs of malnutrition, a history of myocardial infarction, congestive heart failure, renal failure, malignancy, current pneumonia and serum urea level (11). Each variable has its specific score. According to the cumulative score, the predicted 30-day mortality rate is calculated by the formula, and stratified according to the patient's score, which can be divided into three groups: low-, intermediate- and high-risk groups.

### ASAgeCoGeCC score

The ASAgeCoGeCC score stratified hip fracture patients according to their score and assessed their mortality at 30 days, 1 year, 2 years, and 4 years. ASAgeCoGeCC Score consists of age, gender, CCI, ASA score, and cognitive impairment, which shows good calibration and discrimination in predicting early and mid-term mortality after hip fracture in the elderly (12).

### Ships

SHiPS aims to predict 1-year, 3-year and 5-year mortality after hip fracture, whether or not surgery is performed. Based on the Shizuoka Kokuho Database, SHiPS is one of the newly developed risk models for long-term mortality of hip fracture. It uses gender, age, fracture site, nursing certificate, and some comorbidities as variables of the model. Each is assigned 0–9 points, and the total score is between 0 and 64 points. According to the total score, the patients are divided into four risk groups: low, medium, high, and very high, corresponding to 1-year, 3-year, and 5-year death risks, respectively (13).

### Research method

Data collection and evaluation followed a standardized procedure, with all data gathered by two authors (Qiyuan Lu and Houfu Ling) on the same day. Any discrepancies were resolved through group discussion.

By reading the electronic medical records, anesthesia records, surgical records, nursing documents, imaging examinations, laboratory results, etc. of patients who meet the requirements, the variables required for the risk model are collected and recorded retrospectively. Each variable can be found, and then the probability of death risk predicted by each patient in each model is calculated.

Each score was performed by two professional physicians at the same time. The American Society of Anesthesiologists (ASA) classification was obtained from the anesthesia record sheet and evaluated by a senior surgical anesthesiologist. The imaging information was judged by two orthopedic surgeons, and if there were differences, the final judgment was made by another senior orthopedic surgeon. Patient death information was consulted by telephone, and the longest follow-up time was 62 months. This study has been approved by the hospital medical ethics committee and due to the retrospective and observational nature of this study, we do not need to obtain additional personal informed consent.

### Statistical analysis

This study compared the predictive validity of all models using sensitivity, specificity, positive predictive value (PPV), and negative predictive value (NPV). The area under the receiver operating characteristic (AUC) and DeLong test were used to evaluate the ability of the model to distinguish between deceased and surviving patients postoperatively (14). At the same time, Hosmer-Lemeshow goodness-of-fit test and calibration slope (95%CI) were used to evaluate the model calibration (15). All data analysis was performed using R software version 4.4.2 (R Core Team, 2024), and *P* < 0.05 was considered statistically significant.

## Results

### Patient characteristics

From January 2018 to October 2022, after excluding periprosthetic femoral fracture or pathological fracture, a total of 242 elderly patients with fragility hip fractures over 60 years old were treated to our hospital.

**Figure 1:**
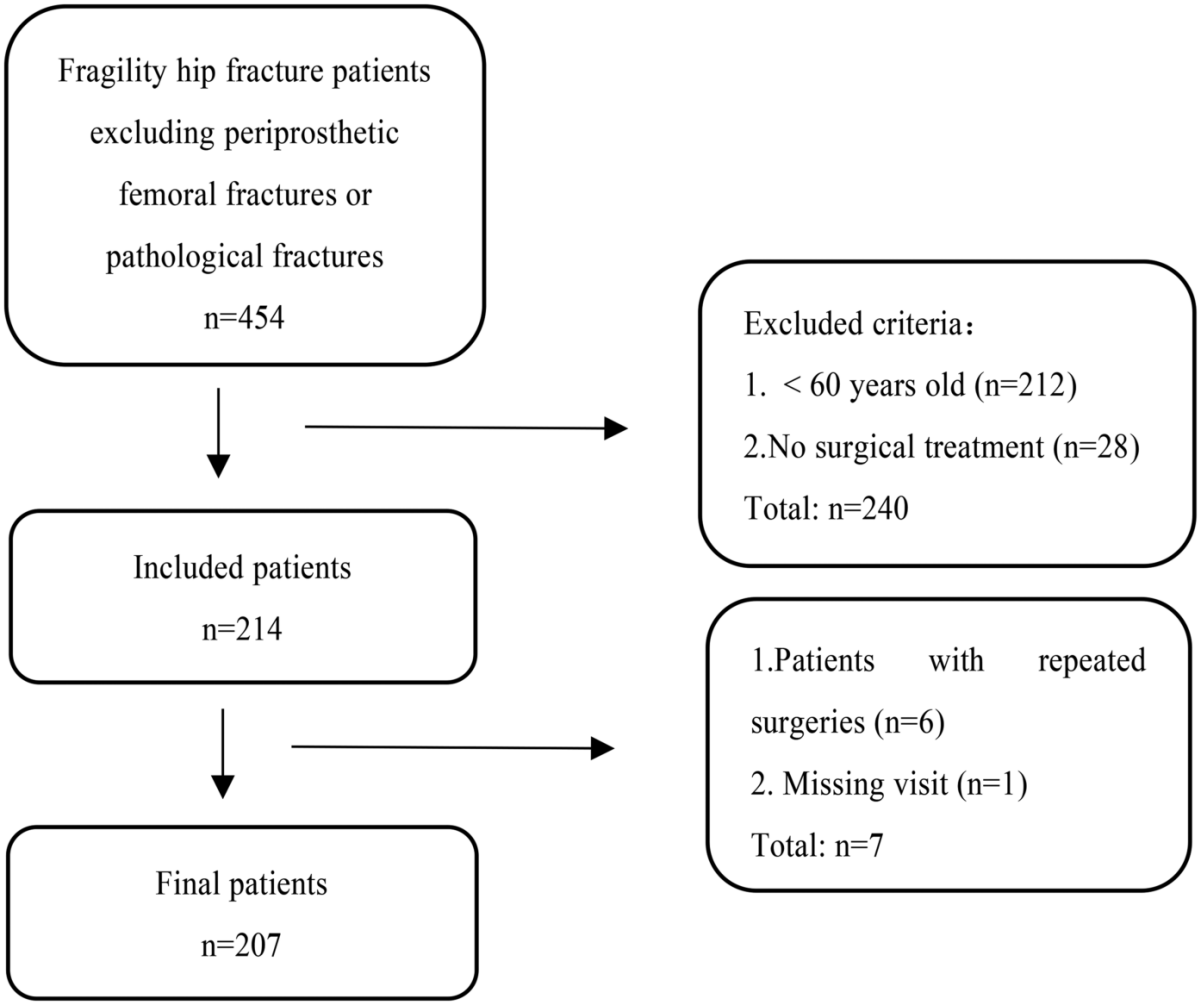
Select the flow chart of the sample patients.

Among them, 214 patients underwent surgical treatment. Of these 214 patients, 6 experienced a contralateral fragility hip fracture within 1 year and were again hospitalised for surgery. For these 6 patients who underwent two surgeries within 1 year, we included their first surgical data into the study. Final analysis included 207 target patients after excluding one lost-to-follow-up case (0.48% attrition rate, below 5%) due to inability to contact either the patient or their family members. The specific flow chart is shown in Figure 1.

The median age of the patients was 83 years old, and most of the patients were female (77.8%). Most of the elderly patients had two or more comorbidities (97.1%), and 91 patients (46.9%) had ASA score >2 (Table 2). The total mortality rate was 29.0% after 1 year.

**Table 2:** Patient characteristics.

Characteristics	*n* (%)
Age	
In years, median (IQR)	83 (71–95)
Gender	
Male	46 (22.2）
Female	161 (77.8)
ASA	
≤S	110 (53.1)
>2	97 (46.9)
Congnitive impariment	
Yes	5 (2.4)
No	202 (97.6)
Pre-fracture mobility	
Independent	159 (76.8)
Dependent	48 (23.2)
Pre-fracture residence	
Home	192 (92.8)
Rehabilitation institution or nursing home	15 (7.2)
Number of comorbidities	
<2	6 (2.9)
≥2	201 (97.1)

IQR, interquartile range.

### Predictive performance

Supplementary Table S1 presents the sensitivity, specificity, PPV and NPV. The Holt et al. demonstrated the highest sensitivity and NPV, whereas the NHFS showed the highest specificity and PPV.

**Figure 2:**
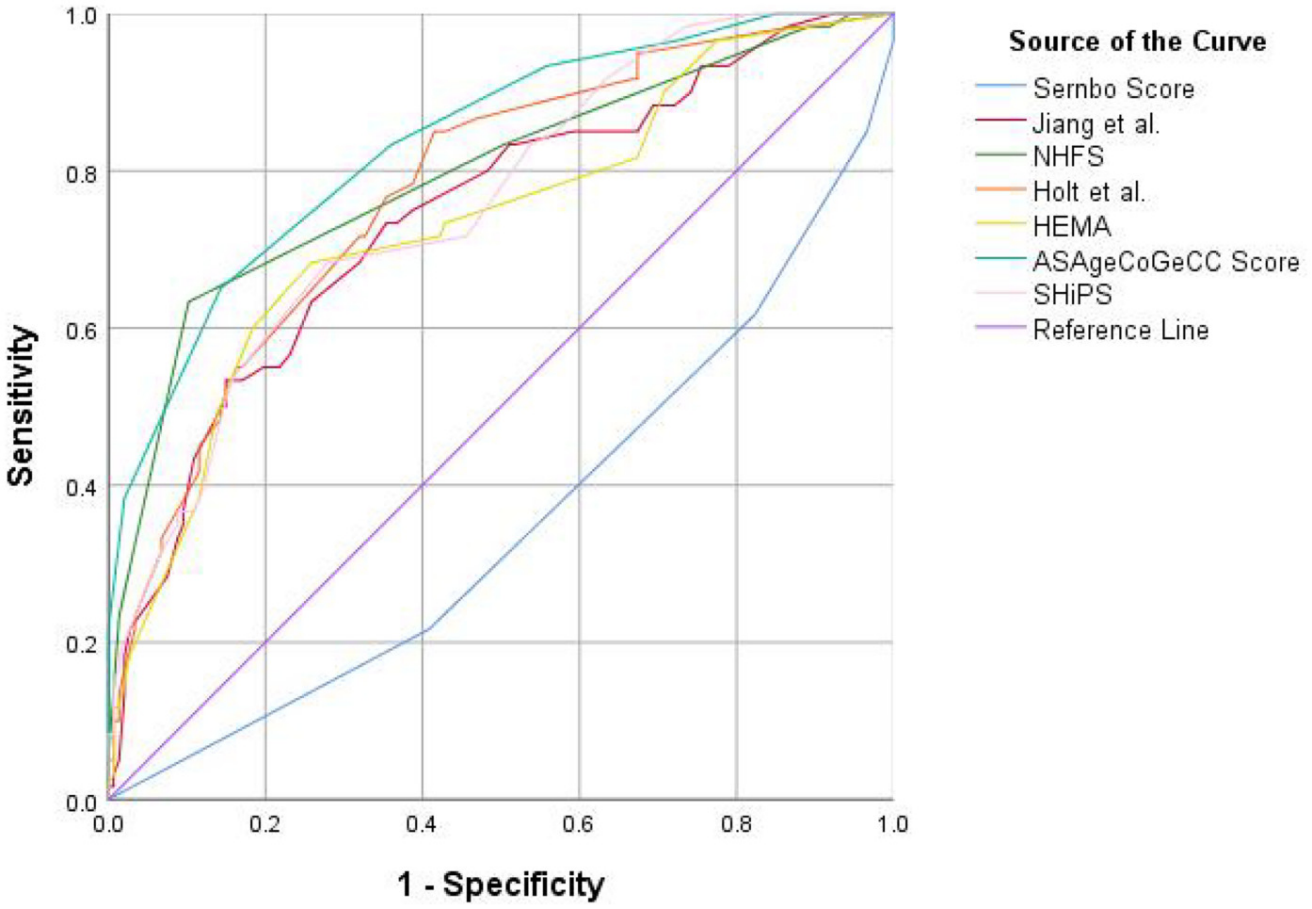
The standard receiver operating characteristic curve (ROC) of 7 death risk prediction models. ROC, receiver operatingcharacteristic; NHFS, Nottingham hip fracture score; HEMA, hip fracture estimator of mortality Amsterdam; SHiPS, Shizuoka Hip Fracture Prognostic Score.

The receiver operating characteristic curve (ROC) and DeLong test were used to show the discrimination of 7 risk prediction models for 1-year mortality. As shown in Figure 2, the ASAgeCoGeCC model (AUC = 0.84, *p* < 0.001) had the best discrimination for 1-year mortality. The NHFS (AUC = 0.80, *p* < 0.001), Holt et al. (AUC = 0.78, *p* < 0.001), SHiPS model (AUC = 0.76, *p* < 0.001) also showed good discrimination. Jiang et al. (AUC = 0.74, *p* < 0.001) and HEMA (AUC = 0.73, *p* < 0.001) had slightly worse prediction ability. The discrimination of Sernbo Score (AUC = 0.35, *p* < 0.001) was not satisfactory, as shown in Table 3.

**Table 3:** The predictive performance of 7 risk prediction models for 1-year mortality in elderly patients with fragility hip fracture.

Risk model	AUC	Hosmer–Lemeshow	Calibration slope (95% CI)
Sernbo Score	0.35 (0.27–0.44), ***p*** **=** **0.001**	*p* = 0.90	0.67 (0.25, 1.10)
Jiang et al.	0.74 (0.67–0.82), ***p*** <** 0.001**	*p* = 0.68	0.89 (0.40, 1.48)
NHFS	0.80 (0.72–0.87, ***p*** <** 0.001**	***p*** **=** **0.02**	2.51 (1.74, 3.38)
Holt et al.	0.78 (0.71–0.84, ***p*** <** 0.001**	*p* = 0.97	1.37 (0.92, 1.88)
HEMA	0.73 (0.66–0.81), ***p*** <** 0.001**	***p*** <** 0.01**	0.51 (0.33, 0.72)
ASAgeCoGeCC Score	0.84 (0.78–0.90), ***p*** <** 0.001**	*p* = 0.36	0.75 (0.48, 1.09)
SHiPS	0.76 (0.69–0.83), ***p*** <** 0.001**	***p*** **=** **0.04**	1.70 (1.11, 2.33)

Values in parentheses are 95% confidence intervals. AUC, area under the ROC curve; NHFS, Nottingham Hip FractureScore; HEMA, hip fracture estimator of mortality Amsterdam; SHiPS, Shizuoka Hip Fracture Prognostic Score. Bold typeface to indicate statistically significant results (*p* < 0.05).

DeLong test results showed that the AUC of ASAgeCoGeCC model was higher than that of HEMA, Jiang et al., SHiPS and Sernbo score (*P* < 0.05), and the Sernbo score showed poor discrimination. See Supplementary Table S2 for details.

The ASAgeCoGeCC, Holt et al., Jiang et al. and Sernbo Score showed good calibration for 1-year mortality, and the results of Hosmer-Lemeshow goodness-of-fit test were not significant (*p* > 0.05), suggesting that the model fitted well, among which the Holt et al. had the best predictive ability (*p* = 0.97), while the NHFS (*p* = 0.02), HEMA (*p* < 0.01) and SHiPS (*p* = 0.04) were obviously lack of fit as shown in Table 3. The calibration slope (95% CI) also showed similar results.

### Risk stratification and accuracy

In addition to the SHiPS divided into low, intermediate, high and very high risk groups, the remaining six risk models are divided into low, intermediate and high risk groups according to cumulative scores. The predicted and observed 1-year mortality rates in the three risk groups are shown in Table 4. Holt et al., SHiPS did not have a corresponding low-risk group in this study. According to the risk stratification, the 1-year estimated mortality rate of the low-risk group was 5.6%, and the actual mortality rate was between 5.8% and 23.4%. The 1-year estimated cumulative mortality rate of the intermediate-risk group was between 1.7% and 18.0%, and the actual mortality rate was between 6.0% and 40.0%; the 1-year mortality risk of the high-risk group was between 5.8% and 50.4%, and the actual mortality rate was between 24.4% and 65.0%.

**Table 4:** Seven mortality risk prediction models risk groups predicted mortality、actual observed mortality and accuracy.

Risk model	Predicted short-term mortality (%)	*N* (%)	Observed 1-year mortality (%)	Accuracy (%)
Low-risk group				
Sernbo score 20 or 17		158 (76.3)	23.4	
Jiang et al. ≤7	12.1	35 (16.9)	11.4	94.2
NHFS ≤3	≤H.7	17 (8.2)	5.9	45.8
Holt et al. <1	<2.2	None	None	
HEMA ≤1	≤E.6	49 (23.7)	12.2	45.9
ASAgeCoGeCC score <7	1.1	69 (33.3)	5.8	19.0
SHiPS 0–9	3.0	None	None	
Intermediate-risk group				
Sernbo score 14		35 (16.9)	40.0	
Jiang et al. 8–21	22.9–36.5	46 (22.2)	13.0	35.6
NHFS 4–5	4.4–6.9	137 (66.2)	15.3	45.1
Holt et al. 1–2	2.2–5.8	50 (24.2)	6.0	96.7
HEMA 1.5–2	8.9–13.9	51 (24.6)	19.6	70.9
ASAgeCoGeCC score 7–8	18.0	78 (37.7)	21.8	82.6
SHiPS 10–17	9.0	62 (30.0)	9.7	92.8
High-risk group				
Sernbo score 11 or 8		14 (6.8)	64.3	
Jiang et al. ≥22	52.0	126 (60.9)	39.7	76.3
NHFS ≥6 (6–9)	≥10.7 (10.7–33.6)	53 (25.6)	71.7	46.9
Holt et al. >2 (2–4.92)	>5.8 (5.8–53.2)	157 (75.8)	36.3	16.0
HEMA ≥2.5 (2.5–8.5)	≥21.0 (21.0–99.1)	107 (51.7)	41.1	41.5
ASAgeCoGeCC score >8	50.4	60 (30.0)	65.0	77.5
SHiPS 18–24	16.0	90 (43.5)	24.4	65.6
SHiPS (very high-risk) 25–64	31.0	55 (26.6)	58.2	53.3

NHFS, Nottingham Hip FractureScore; HEMA, Hip fracture estimator of mortality Amsterdam; SHiPS, Shizuoka Hip Fracture Prognostic Score.

We conducted a comprehensive comparison of 7 models in the Supplementary Table S3.

**Figure 3:**
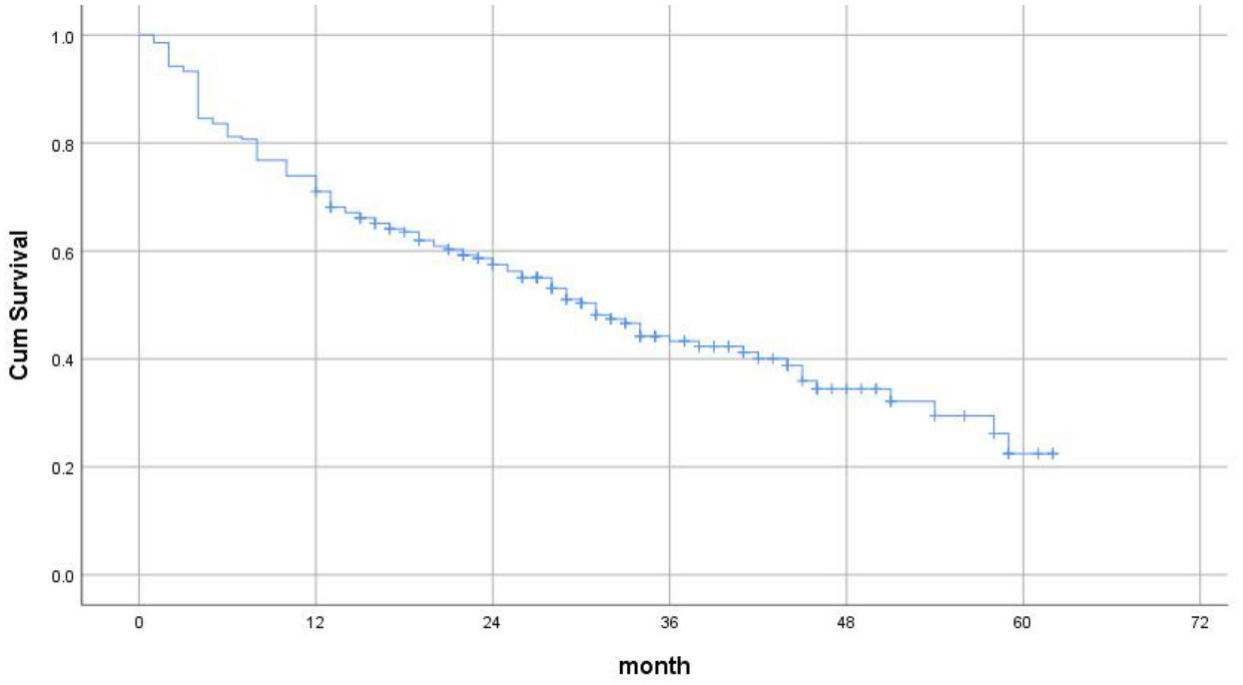
Kaplan–Meier survival analysis.

Figure 3 shows the Kaplan–Meier Survival Analysis of elderly patients with fragility hip fractures after surgery in this study. The longest follow-up time was 62 months, and the risk of death was the highest at 1 year after surgery.

## Discussion

The present study documented a 29.0% 12-month postoperative mortality rate among geriatric fragility hip fracture patients, falling within the established literature range of 13.4 1 year (16, 17). Kaplan-Meier survival analysis revealed the highest mortality risk occurring within the first postoperative year (*P* < 0.001), underscoring this temporal window as a critical intervention period for mortality reduction (5).

The analysis of predictive validity, discrimination, and calibration suggests that the ASAgeCoGeCC Score may be the only model demonstrating balanced sensitivity (0.73)/specificity (0.82), excellent discrimination, and good calibration. The NHFS and Holt et al. ranked second, while the Sernbo Score showed relatively poor predictive performance. This finding contrasts with previous studies which reported satisfactory predictive performance for the Sernbo Score (18).

The ASAgeCoGeCC Score is mainly based on the Age-adjusted Charlson Comorbidity Index (aCCI) and consists of several risk factors with strong correlation after fragility hip fracture surgery in the elderly, but does not focus on patients under 65 years with aCCI of 2 points, which may need further adjustment. As one of the most commonly used mortality risk models for hip fractures, NHFS has been repeatedly verified for its predictive efficacy for 30 days, 1 year or longer (19–21). Holt et al. showed satisfactory predictive ability in both this study and external validation (21, 22). It subdivides the ASA score, focuses on the type of fracture, and considers pathological fractures, which most models do not have. SHiPS has shown good predictive performance for the early and mid-term mortality risk of hip fracture patients with or without surgery. The model clarifies the degree of increase in mortality caused by each comorbidity, which is a novelty. The predictive performance in this study is second only to Holt et al. In previous studies, the model AUC of Jiang et al. was between 0.74 and 0.78, showing acceptable predictive performance (8, 22). It subdivides comorbidities and pays attention to the impact of cardiovascular and respiratory diseases on elderly patients. HEMA showed good predictive ability in the development set and validation set (AUC = 0.79–0.81) (11). However, the results are not ideal in external verification (21). In addition to the lack of strong correlation variables and small sample size, the model is too complex is also one of the reasons (23).

It should be noted that in this study, an adaptation was made to the NHFS: we used a history of cognitive impairment instead of the Abbreviated Mental Test Score (AMTS). This modification shifted the nature of the evaluation from a strict external validation to an assessment of an adapted model. The value of this approach lies in preventing the exclusion of a substantial number of cases due to missing data while enhancing clinical utility.

Due to the limitation of sample size and number of events, this study only makes an exploratory analysis of the risk model. It may not be reasonable to abandon the model with strong discrimination just because the calibration is not ideal (24). The observed performance discrepancies in this study may arise from population, temporal, or variable-related differences: 1. Population Heterogeneity: The baseline characteristics, healthcare practices, or postoperative care standards differed between the original model development cohorts and our study population. For instance, HEMA was developed using Dutch populations, whereas this study focused on individuals from Southern China; 2. Temporal Mismatch: HEMA was originally designed to predict 30-day mortality, while our study examined 1-year outcomes, potentially introducing calibration bias due to temporal extrapolation; 3. Variable Measurement Discrepancies: Retrospective data collection may have led to inaccuracies in recording critical variables (e.g., cognitive status in NHFS) or inconsistencies in measurement protocols compared to the original model definitions (e.g., subjectivity in ASA grading or diagnostic criteria for comorbidities in SHiPS). These factors could degrade predictive accuracy and cause systematic deviation between predicted probabilities and actual risks.

The Sernbo Score demonstrated inadequate mortality prediction capacity. NHFS, Holt et al., and HEMA mainly target 30-day mortality, which may explain the relatively low accuracy of risk stratification in this study. Among the seven models, Jiang et al.'s algorithm achieved superior predictive precision in low-risk stratification while maintaining high-risk group accuracy, albeit with significant mortality overestimation in intermediate-risk categories. Conversely, the ASAgeCoGeCC Score showed high validity in intermediate- and high-risk groups but overestimated mortality in low-risk patients, potentially attributable to miscalibration of patients aged <65 years with aCCI = 2. SHiPS demonstrated 53.3 53.33.3s with aCCIears with aCCIgh-risk groups but overestimated mortality in low-risk patients, potentially attributable to t mortalit

Our cohort lacked low-risk patients as defined by the Holt et al. and SHiPS models. This absence limits their applicability, preventing identification of true low-risk individuals and impairing their triage function, thereby reducing their value for tiered care. Furthermore, the incomplete representation across risk strata compromises stratification completeness, leading to fragmented risk assessment that hinders clinical decisions. Critically, patients potentially misclassified due to the missing low-risk stratum could receive overly intense interventions, creating a treatment-risk imbalance that undermines the models’ generalizability in this population.

Notably, despite proliferating hip fracture prediction models, no consensus exists regarding optimal high-quality mortality risk prediction model for geriatric fragility hip fracture populations.

Nitchanant Kitcharanant et al.'s machine learning-derived model (Thailand) showed preliminary predictive validity but suffered from methodological limitations including single-center recruitment, inadequate sample power, and absence of risk stratification—factors precluding inclusion in our comparative analysis. Nevertheless, their computational approach presents novel management paradigms (25).

NHFS has repeatedly shown its excellent performance in predicting early mortality after hip fracture surgery in external validation. A review incorporating the prediction models of hip fracture before 2019 recommended NHFS as the first choice at admission (26). For patients with fragility hip fractures, Takawira C Marufu et al.suggested that NHFS is a simple, inexpensive, easy to calculate, objective and accurate tool for assessing perioperative morbidity and mortality compared to 25 risk stratification tools such as ASA and CCI. And may be the most appropriate of the currently available score (18). In our study, the NHFS also showed satisfactory predictive performance.

The risk model is based on risk factors associated with adverse outcomes after hip fracture surgery. Among them, age, male, treatment method, operation time, ASA score, comorbidity or high CCI point, walking ability before fracture, cognitive impairment were the most common (16, 27–30). Age, male, comorbidity, surgical treatment, and anti-osteoporosis treatment are considered to be risk factors that are strongly associated with the risk of death from fragility hip fractures (16, 26, 31, 32).

The additional mortality shown by different subgroups in this study suggests that the risk of death in patients may also be related to unknown factors that are not included in these models. If it is unrealistic to develop a mortality risk model that includes all the risk factors that have been studied as variables, there are unexplained additional mortality rates even after adjustment for these factors, and may sacrifice clinical applicability (33). Both predictive ability and clinical applicability are important. For patients considering emergency surgery, the model should be simple and easy, while for patients undergoing elective surgery, the model can be relatively more complex.

The elevated mortality associated with fragility hip fractures in geriatric populations underscores the critical role of anti-osteoporosis pharmacotherapy (32, 34). Osteoporotic therapeutic interventions have been demonstrated to confer dual therapeutic benefits: mortality reduction in elderly fragility hip fracture patients, coupled with mitigation of secondary fracture risk (32, 34–36) and enhanced postoperative functional recovery (37, 38).

Notwithstanding the lower incidence of fragility hip fractures in male populations compared to female counterparts (32), male populations demonstrates significantly elevated mortality rates. In this study, compared with women, the median age of male patients was 13.5 years younger than that of women. The 1-year mortality rate was 52.2% in men and 22.4% in women. Longitudinal follow-up confirmed persistent male mortality predominance (*p* < 0.01). Multiple etiological factors underlie this disparity, with two predominant determinants identified: (1) systematic underdiagnosis of osteoporosis in male populations, and (2) suboptimal therapeutic adherence patterns in male patients (39).

The high mortality rate after surgery not only suggests the importance of anti-osteoporosis treatment, but also shows the importance of postoperative nursing and rehabilitation. In terms of etiology, more than 90% of hip fractures in the elderly are associated with falls (40). Improving personal health and addressing unsafe external factors can help prevent falls (41).

There is no doubt that surgical treatment is still the gold standard for the treatment of fragility hip fractures in the elderly (42). The most common causes of death after hip fractures are directly related to fracture or surgery, infection, and a series of subsequent adverse events (16). Two major causes of death are preventable: pneumonia and decreased function (28, 43).

In addition to the above general requirements, risk stratification makes postoperative care and rehabilitation more targeted (32). The treatment provided by family or institutional care does not effectively improve the functional prognosis of all patients, and it is unrealistic for all patients to receive the same level of rehabilitation care due to economic factors and social burdens.

For the low and intermediate risk groups, after surgery, they can participate in a more proactive rehabilitation nursing plan, get better functional results, restore independence as soon as possible, and improve the quality of life. For the high risk group, it may require careful preoperative comorbidity management and surgical timing optimization, follow the principle of individualization, choose a more appropriate anesthesia and surgical plan, and postoperative management needs to be more systematic, and even requires multidisciplinary participation. Different risk groups guide the selection of different rehabilitation and nursing programs, from home care to rehabilitation institutions and even multidisciplinary intervention. The ultimate goal is to achieve better economic and social benefits and improve the quality of life of patients. For patients with low and intermediate risk of fragility hip fracture, we hope that they can return to independent life or life before fracture, improve bone mineral density, and enhance daily activity ability. For high risk patients, we hope to reduce the possibility of recurrent fragility fracture, increase their clinical life, and improve the quality of follow-up life. The mortality risk prediction model can be used as a useful tool.

By evaluating the predictive performance of the model, the advantages of this study lie in the following four points: First, it points out the high mortality rate of elderly patients with fragility hip fractures. Second, elderly male patients with fragility hip fractures should receive more attention. Third, suggestions for reducing postoperative mortality were proposed. Fourth, the overall rehabilitation and nursing requirements after surgery were proposed and detailed clinical guidance was performed according to the risk stratification. Fifth, at present, there is no recognized good and accurate high-quality mortality risk prediction model for elderly patients with fragility hip fractures. Our study attempts to provide new ideas for the diagnosis and treatment of such patients in clinical practice, and to arouse the attention of enhancing the management of elderly patients with fragility hip fractures.

This retrospective study has several limitations: primarily, the sample size and number of event outcomes are insufficient. Based on literature and model performance assumptions (anticipated AUC = 0.70, 95% CI width ±0.05; acceptable calibration slope bias ±0.15; assumed 1-year mortality 13.4–30.0%, *α* = 0.05 two-sided), the Hanley & McNeil formula estimated requiring a minimum total sample size of 1,076. However, this study only included 207 cases (19.2% of the theoretical requirement). This leads to: (1) significantly reduced precision in AUC estimation, such as the ASAgeCoGeCC model's AUC = 0.84 having a 95% CI width reaching 0.14 (which should be <0.06 with sufficient samples), being 133% lower than theoretical precision; (2) reduced reliability due to excessively wide confidence intervals for calibration slopes (e.g., the Holt model's 1.37 having a 95% CI width of 0.97, while sufficient samples should yield <0.4); (3) decreased statistical power for model comparisons, such as ASAgeCoGeCC vs. HEMA where calculated power is only 68% (recommended ecommen(4) an events-to-predictors ratio of merely 8.6:1 (recommended mended yield <0.4); slopes (e.g., the Holt modeltheoretical precision; d 207 cases (19.2% of the theoretical requieading to predictive accuracy being overestimated or underestimated (44), which may partially explain the inter-model accuracy differences observed in this study. As a preliminary exploratory analysis, this study has no absolute sample size requirements; however, this similarly affects the precision and reliability of conclusions.

Second, some modelslly explain theSernbo Score, Holt et al., and HEMA—were not originally designed to predict 1-year mortality after hip fracture surgery. Temporal extrapolation of these models may limit variable applicability, as the exclusion of chronic diseases or socioeconomic factors influencing long-term mortality omits critical predictors (45). Although these models demonstrated good calibration within 30 days, shifts in baseline risks during long-term prediction (e.g., new-onset medical conditions) may decouple predicted probabilities from observed mortality rates, causing calibration drift (46). This could also degrade statistical performance: discriminative ability (AUC) may decline due to unaccounted long-term risk factors, while overfitting risks and inaccurate risk stratification emerged, aligning with the observed performance of these models in our study.

Third, as a retrospective study, inherent methodological limitations and potential biases are unavoidable. Selection bias primarily stems from the single-center sample's limited representativeness, potentially leading to an underestimation of the true mortality rate; loss to follow-up could further underestimate risk. Information bias includes reliance on medical record-based predictor variables and potential misclassification of retrospectively ascertained outcomes, which may underestimate the model's performance. Furthermore, unmeasured confounding factors (e.g., social support) might affect model accuracy and obscure the causal relationship between the risk score and mortality. Additionally, the small sample size and overfitting could artificially inflate the apparent performance of some models.

These limitations may compromise predictive validity, restrict generalizability, and reduce clinical utility due to residual confounding. While exploratory analysis was performed, future improvements require prospective designs, multicenter collaborations, or shared public databases to expand sample sizes, develop dynamic scoring systems, and conduct rigorous external validation to balance scientific rigor with clinical feasibility (44).

## Conclusion

To our knowledge, this is one of the few studies to externally evaluate seven models for predicting 1-year postoperative mortality risk in older patients with fragility hip fractures within a single Chinese cohort. The ASAgeCoGeCC Score demonstrated a sensitivity/specificity of 0.73/0.82, an AUC of 0.84, and calibration analysis including a Hosmer-Lemeshow *p*-value of 0.36 and a calibration slope of 0.75, indicating robust predictive performance. The NHFS and Holt et al. performed next best. The ASAgeCoGeCC Score and NHFS both seem to be easy to use, but NHFS has been externally verified many times. The SHiPS showed the highest accuracy in risk stratification and seemed to be more clinically applicable. Further studies are needed to verify the above conclusions. Further model studies on the elderly population with fragility hip fractures are needed in the future and determine the best risk model for predicting postoperative mortality (14).

## Data Availability

The raw data supporting the conclusions of this article will be made available by the authors, without undue reservation.
