# Supplementary material for: Prediction of 1-year post-operative mortality in elderly patients with fragility hip fractures in China: evaluation of risk prediction models
Source: Front Surg. 2025 Jun 23;12:1415680. doi: 10.3389/fsurg.2025.1415680 (PMC12230039; doi:10.3389/fsurg.2025.1415680)
Supplement: Supplementary file 1 [file Datasheet1.pdf]

Supplementary Table 1 The predictive validity of 7 risk prediction models for 1-year mortality in elderly patients with fragility hip fracture.

Risk model	Sensitivity	Specificity	PPV	NPV
Sernbo Score	0.38	0.82	0.46	0.76
Jiang et al.	0.53	0.85	0.59	0.82
NHFS	0.63	0.90	0.72	0.86
Holt et al.	0.85	0.59	0.46	0.91
HEMA	0.68	0.74	0.52	0.85
ASAgeCoGeCC Score	0.73	0.82	0.62	0.88
SHiPS	0.68	0.72	0.50	0.85

NHFS: Nottingham Hip FractureScore;*HEMA*:Hip fracture estimator of mortality Amsterdam;*SHiPS*:Shizuoka Hip Fracture Prognostic Score; PPV, positive predictive value; NPV, negative predictive value.

Supplementary Table 2 Combined results of DeLong tests for the 7 risk models.

Comparative model group	AUC difference (95% CI)	Z	p
NHFS vs. HEMA	0.062(-0.027, 0.151)	1.367	0.172
NHFS vs. Jiang et al.	0.054(-0.037, 0.145)	1.160	0.246
NHFS vs. ASAgeCoGeCC Score	-0.051(-0.123, 0.020)	-1.413	0.158
NHFS vs. Holt et al.	0.020(-0.051, 0.091)	0.554	0.580
NHFS vs. SHiPS	0.039(-0.029, 0.106)	1.122	0.262
NHFS vs. 1/Sernbo Score	0.148(0.075, 0.220)	3.989	**＜0.001**
HEMA vs. Jiang et al.	-0.008(-0.042, 0.025)	-0.476	0.634
HEMA vs. ASAgeCoGeCC Score	-0.113(-0.184, -0.042)	-3.125	**0.002**
HEMA vs. Holt et al.	-0.042(-0.142, 0.058)	-0.817	0.414
HEMA vs. SHiPS	-0.023(-0.102, 0.055)	-0.582	0.561
HEMA vs. 1/Sernbo Score	0.086(-0.017, 0.189)	1.638	0.101
Jiang et al. vs. ASAgeCoGeCC Score	-0.105(-0.173, -0.037)	-3.026	**0.002**
Jiang et al. vs. Holt et al.	-0.034(-0.128, 0.061)	-0.698	0.485
Jiang et al. vs. SHiPS	-0.015(-0.092, 0.061)	-0.389	0.697
Jiang et al. vs. 1/Sernbo Score	0.094(-0.009, 0.197)	1.793	0.073
ASAgeCoGeCC Score vs. Holt et al.	0.071(0, 0.143)	1.950	0.051
ASAgeCoGeCC Score vs. SHiPS	0.090(0.026, 0.154)	2.738	**0.006**
ASAgeCoGeCC Score vs. 1/Sernbo Score	0.199(0.115, 0.284)	4.617	**＜0.001**
Holt et al. vs. SHiPS	0.018(-0.051, 0.088)	0.519	0.604
Holt et al. vs. 1/Sernbo Score	0.128(0.068, 0.188)	4.167	**＜0.001**
SHiPS vs. 1/Sernbo Score	0.109(0.035, 0.183)	2.893	**0.004**

NHFS Nottingham Hip FractureScore. *HEMA* Hip fracture estimator of mortality Amsterdam. *SHiPS* Shizuoka Hip Fracture Prognostic Score

Supplementary Table 3 A comprehensive comparison of 7 models: variables included, ease of use, AUC, calibration, external validation, and clinical applicability.

Risk model	Variables	Ease of use	AUC	Hosmer–Lemeshow	External validation	Clinical applicability
**Sernbo Score**	Age<br>Habitat<br>Walking aids<br>Mental status	Simple	0.35	p=0.90	Yes	Suitable for rapid risk assessment and resource-constrained environments
**Jiang et al.**	Age<br>Male sex<br>Admitted from long term care<br>COPD<br>Pneumonia<br>Ischemic heart disease<br>Previous myocardial infarction<br>Any cardiac arrhythmia<br>Congestive heart failure<br>Malignancy<br>Malnutrition<br>Any electrolyte disorder<br>Renal failure	Medium	0.74	p=0.68	There is no independent external validation report.	Suitable for identifying high-risk patients and guiding peri-operative resource allocation.
**NHFS**	Age in years	Simple	0.80	p=0.02	Yes	The core advantage is that it is

	Sex<br>Admission Hb<br>Cognitive impairment<br>Living in an institution<br>Number of co-morbidities<br>Malignancy					fast, simple, and does not require additional testing. It is suitable for primary hospitals or environments with limited resources, such as emergency triage or perioperative resource optimization.
**Holt et al.**	Age in years<br>ASA score<br>Gender<br>Pre-fracture residence<br>Pre-fracture mobility<br>Fracture type	Difficult	0.78	p=0.97	There is no independent external validation report.	It is suitable for a well-resourced medical system to promote early surgical intervention and identify high-risk patient groups.
**HEMA**	Age in years<br>In-hospital fracture<br>Signs of malnutrition<br>Myocardial infarction<br>Congestive heart failure<br>Current pneumonia<br>Renal disease<br>Malignancy<br>Serum urea	Medium	0.73	$p<0.01$	There is no independent external validation report.	It is suitable for a well-resourced medical system, paying attention to both short-term and long-term prognosis, and providing management direction for intervention indicators (such as nutrition and anemia).

**ASAgeCoGeCC Score**	Age<br>CCI<br>Cognitive impairment<br>ASA<br>Gender	Simple	0.84	p=0.36	There is no independent external validation report.	It is fast and simple, and is suitable for clinical environments with limited resources or rapid decision-making.
**SHiPS**	Sex<br>Age<br>Fracture site<br>Nursing care certification<br>Comorbidity	Medium	0.76	p=0.04	There is no independent external validation report.	Localized, socially sensitive tools for doctor-patient co-development of treatment goals (e.g., surgery vs.conservative treatment) and long-term care planning.

NHFS Nottingham Hip FractureScore. *HEMA* Hip fracture estimator of mortality Amsterdam. *SHiPS* Shizuoka Hip Fracture Prognostic Score
